# Reliability and Validity of the Quick Inventory of Depressive Symptomatology—Self-Report Scale in Older Adults With Depressive Symptoms

**DOI:** 10.3389/fpsyt.2021.686711

**Published:** 2021-10-20

**Authors:** Rui Liu, Fei Wang, Shou Liu, Qinge Zhang, Yuan Feng, Kang Sim, Xiling Cui, Jing-Xia Lin, Gabor S. Ungvari, Yu-Tao Xiang

**Affiliations:** ^1^The National Clinical Research Center for Mental Disorders, Beijing Key Laboratory of Mental Disorders, Beijing Anding Hospital, The Advanced Innovation Center for Human Brain Protection, Capital Medical University, Beijing, China; ^2^Guangdong Mental Health Center, Guangdong Provincial People's Hospital, Guangdong Academy of Medical Sciences, Guangzhou, China; ^3^Department of Public Health, Medical College, Qinghai University, Xining, China; ^4^West Region, Institute of Mental Health, Singapore, Singapore; ^5^Department of Business Administration, Hong Kong Shue Yan University, Hong Kong, Hong Kong, SAR China; ^6^Department of Rehabilitation Sciences, Hong Kong Polytechnic University, Hong Kong, Hong Kong, SAR China; ^7^Department of Psychiatry, University of Notre Dame Australia, Fremantle, WA, Australia; ^8^Division of Psychiatry, School of Medicine, University of Western Australia/Graylands Hospital, Perth, WA, Australia; ^9^Unit of Psychiatry, Department of Public Health and Medicinal Administration, Institute of Translational Medicine, Faculty of Health Sciences, University of Macau, Macau, Macao, SAR China; ^10^Centre for Cognitive and Brain Sciences, University of Macau, Macau, Macao, SAR China; ^11^Institute of Advanced Studies in Humanities and Social Sciences, University of Macau, Macau, Macao, SAR China

**Keywords:** depression, older adults, reliability, Quick Inventory of Depressive Symptomatology—Self-Report, validity

## Abstract

**Background:** Depressive symptoms are common in older adults. Developing rapid self-report tools is essential to measure the presence and severity of depressive symptoms in older adults. This study evaluated the psychometric properties of the Quick Inventory of Depressive Symptomatology—Self-Report (QIDS-SR) scale for use in depressed older adults.

**Methods:** A total of 238 depressed older adults were included in the study. The Montgomery–Asberg Depression Rating Scale (MADRS) and the QIDS-SR were administered to assess the severity of depressive symptoms. Cronbach's alpha coefficient, Spearman rank correlations, and principal component analysis were performed to estimate the internal consistency, convergent validity, and factorial structure of the QIDS-SR, respectively.

**Results:** The Cronbach's alpha for the QIDS-SR was acceptable (α = 0.64). Item–total correlation analyses showed that the items of concentration/decision-making, involvement, energy level, and agitation/retardation had high correlation with the QIDS-SR total score (all correlation coefficients ≥0.60). The QIDS-SR total score was significantly correlated with the MADRS total score (*r* = 0.53, *p* < 0.001), demonstrating acceptable convergent validity. Factor analysis revealed the unidimensional structure of the QIDS-SR.

**Conclusions:** The QIDS-SR appears to be a reliable and valid self-report scale for estimating the severity of depressive symptoms in depressed older adults.

## Introduction

Depressive symptoms (depression thereafter) are common in older adults (1–3). With the rapid aging of the population in many countries, depression in older adults has become a great public health challenge globally, particularly in developing countries. The prevalence of depression measured with the Geriatric Depression Scale (GDS) was 16% in older adults in China (4), and the corresponding figure assessed with the GDS was up to 33% in hospitalized older adults with medical conditions (5). Depression is associated with increased risk of medical co-morbidities, impaired cognitive and social functioning, poor quality of life, and suicide (6–8). Depression is difficult to identify in older adults due to overlapping symptoms with features of normal aging, such as sleep disturbances and poor appetite (9, 10). Thus, developing quasi-accurate and time-efficient instruments to measure older adults' depression is important for identifying this debilitating illness that negatively impacts the quality of life of sufferers.

Several clinician-rated scales have been developed for measuring the presence and severity of depression, such as the Hamilton Rating Scale for Depression (HAMD) (11) and the Montgomery–Asberg Depression Rating Scale (MADRS) (12). The two main limitations for clinician-rated scales on depression in general and older adults in particular are that they are time-consuming and require psychiatrically trained interviewers (13). In contrast, self-report scales on depression are time-effective, cheap, and useful and can produce results similar to those obtained by clinician-rated scales (14, 15). For these reasons, a number of self-report scales on depression have been developed to meet the needs of clinical practice and research, such as the GDS (16), the Centre for Epidemiology Studies Depression Scale (CES-D) (17), and the Beck Depression Inventory (BDI) (18).

The 16-item Quick Inventory of Depressive Symptomatology–Self-Report (QIDS-SR) is another widely used self-report instrument covering depressive symptoms incorporating nine Diagnostic and Statistical Manual of Mental Disorder-IV (DSM-IV) diagnostic criteria for major depressive disorders (19). The original version of the QIDS-SR was validated in the elderly in the USA (20). The Chinese version of the QIDS-SR has good psychometric properties in depressed adult patients co-morbid with schizophrenia (21) and hepatitis B virus (HBV) infection (22) but not in older adults. There is compelling evidence that clinical features of depression are considerably determined by sociocultural factors (23, 24). Therefore, findings obtained in the Western socio-cultural context cannot be generalized to other parts of the world with a variety of different socioeconomic backgrounds.

There are ~250.6 million adults aged 60 years or older in China (25), and this population will reach 450 million by 2050 (26). Considering the common occurrence of depression in older adults (4), validating a self-report measure such as the QIDS-SR has great clinical utility for the identification of depression in China. To this end, this study examined the reliability and validity of the QIDS-SR in depressed old Chinese adults.

## Methods

### Settings and Subjects

This study was conducted in three public nursing homes located in Qinghai and Guangdong provinces, China. All residents who met the following entry criteria participated in the study: they were aged ≥60 years, had a total score of MADRS of 7 or above in an interview by a research psychiatrist, could speak and understand the Mandarin dialect of Chinese, understood the aims of the study, and provided written informed consent. Residents with evident cognitive impairment or current major depressive episode were excluded from the study based on a review of their health records by a research psychiatrist. The study protocol was approved by the Institutional Review Board of the University of Macau.

### Assessment

Participants' basic demographic and clinical characteristics were recorded in a data sheet designed for this study. The Chinese version of the QIDS-SR was used to measure the severity of depressive symptoms in the following nine domains during the past week: (1) sad mood, (2) concentration/decision-making, (3) self-outlook, (4) thoughts of death or suicide, (5) involvement, (6) energy level, (7) sleep (i.e., the highest score on any one of the four relevant items—onset insomnia, mid-nocturnal insomnia, early morning insomnia, and hypersomnia), (8) appetite/weight change (i.e., the highest score on any one of the four relevant items—weight increase and decrease and appetite increase and decrease), and (9) agitation/retardation (i.e., the highest score on any one of the two relevant items—psychomotor slowing or psychomotor agitation) (19, 27). The QIDS-SR total score ranges from 0 to 27, with a higher score indicating more severe depressive symptoms (19, 27).

The Chinese version of the 10-item MADRS was the comparator rating instrument to assess the severity of depression within the past week (12, 28). Each item of the MADRS is scored from 0 to 6, and thus the total score ranges from 0 to 60, with a higher score indicating more severe depression (12). The MADRS has satisfactory psychometric properties in depressed Chinese patients (28, 29). The MADRS was rated by a research psychiatrist blind to the QIDS-SR scores. The QIDS-SR assessment was conducted first, followed by the MADRS assessment.

### Statistical Analysis

Data were analyzed using the SPSS, Version 24.0 (IBM SPSS, IBM Crop., Armonk, NY, USA). Internal consistency was examined with the Cronbach's alpha coefficient; an alpha of 0.6 or higher was considered acceptable (21, 30). The item–total correlations of the QIDS-SR were calculated using Spearman rank correlations. Convergent validity was assessed with the Spearman rank correlations between the QIDS-SR and MADRS total scores. The dimensionality of the QIDS-SR was examined using exploratory factor analysis. A principal component analysis (PCA) with Varimax rotation was performed to extract the factors and obtain the most meaningful original factor structure of the QIDS-SR. If one factor explained 20% or more of the total variance, the scale was regarded unidimensional (21, 31, 32). The significance level was set to <0.05 (two-tailed).

## Results

A total of 238 older adults fulfilled the entry criteria and were included in this study. The participants' basic demographic and clinical characteristics are summarized in Table 1. The mean age was 79.13 [standard deviation (SD): 8.16] years; men accounted for 39.08% of the sample, and the mean MADRS score was 15.71 (SD: 5.96).

**Table 1:** Basic demographic and clinical characteristics of the study sample.

	**Study sample** (***n*** **= 238)**	
	**N**	**%**
Male gender	93	39.08
Married/cohabitating	56	23.53
Secondary school or above	89	37.39
Smoking	44	18.49
Religious affiliation/beliefs	131	55.04
Financial status		
Good	112	47.06
Fair	92	38.66
Poor	34	14.29
Family history of psychiatric disorders	4	1.68
Ongoing medical conditions	216	90.76
	**Mean**	**SD**
Age (years)	79.13	8.16
Number of major medical conditions	2.87	1.77
MADRS total	15.71	5.96

*MADRS, Montgomery–Asberg depression rating scale*.

**Figure 1:**
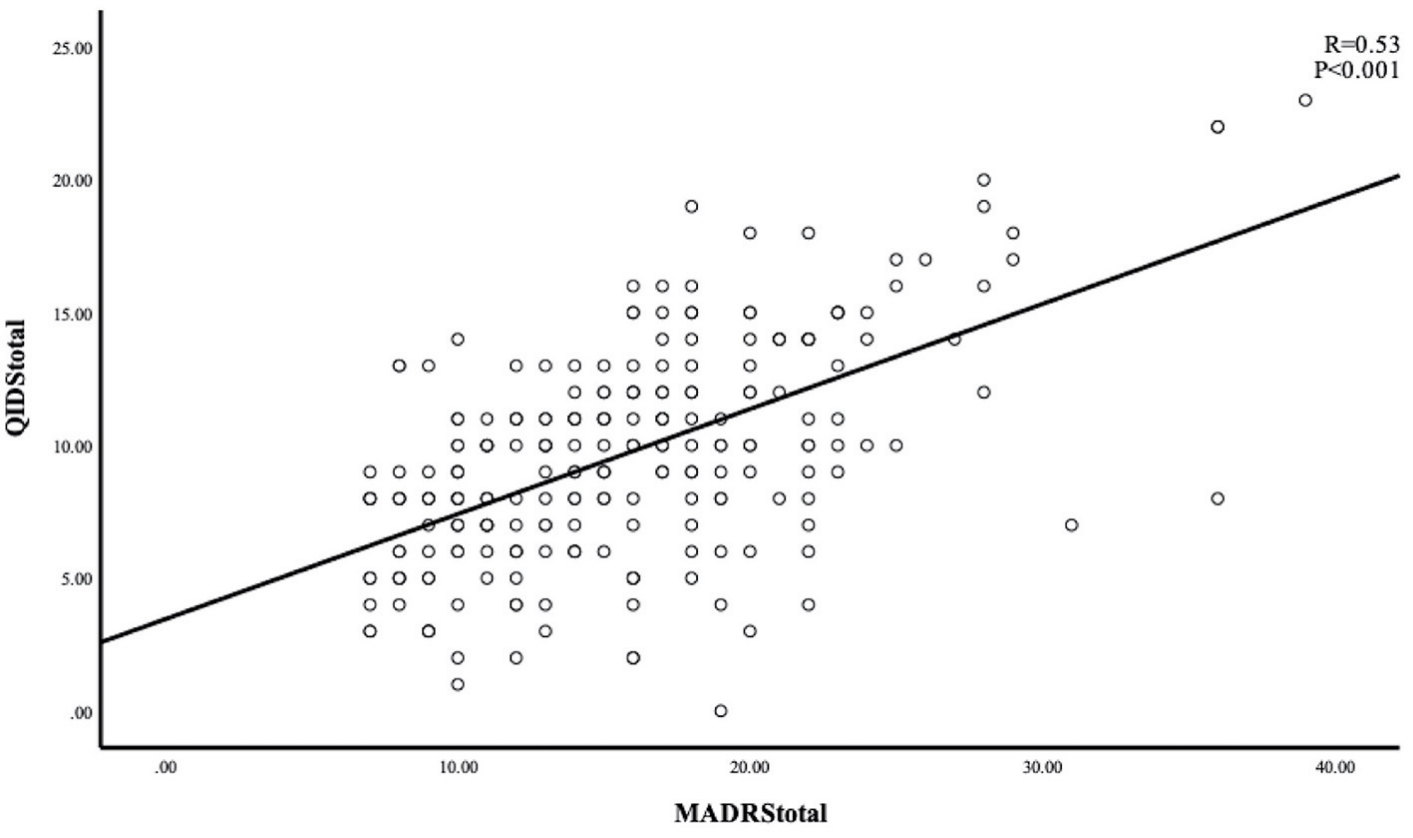
Scree plot for the QIDS-SR at baseline.

**Figure 2:**
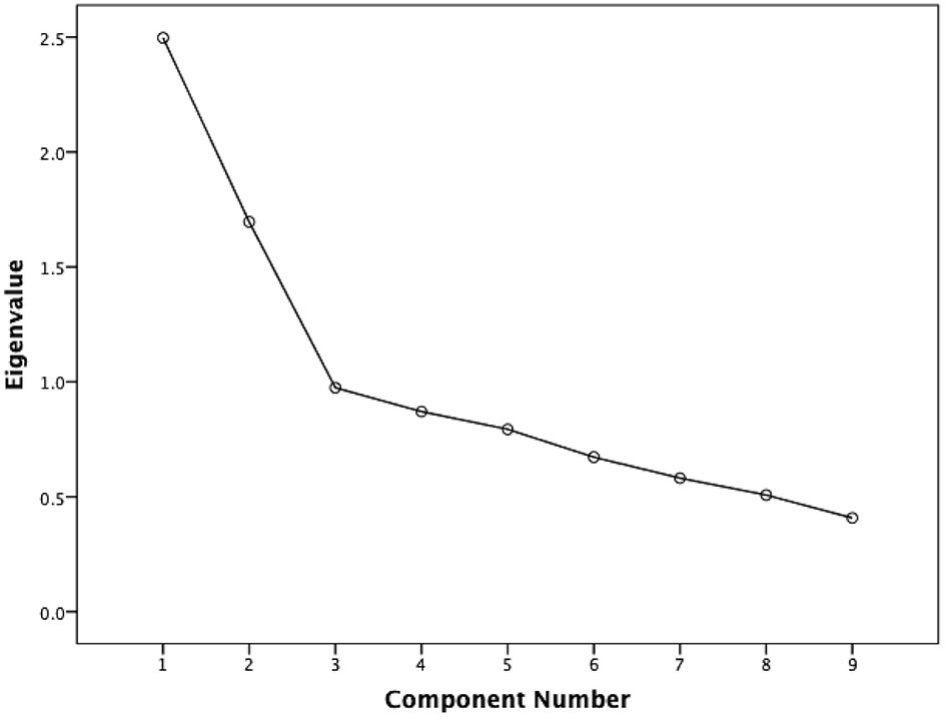
Scatterplot between individual MADRS and QIDS-SR scores. MADRS, Montgomery–Asberg depression rating scale; QIDS-SR, quick inventory of depressive symptomatology—self-report.

The Cronbach's alpha 0.64 showed an acceptable internal consistency and homogeneity between the QIDS-SR items. Four domains (concentration/decision-making, involvement, energy level, and agitation/retardation) had high correlations with the QIDS-SR total score (all correlation coefficients ≥0.60) (Table 2). The correlation coefficient between the QIDS-SR and the MADRS was 0.53 (*p* < 0.001), indicating acceptable convergent validity (Figure 1). Figure 2 shows the scree plots with magnitude of eigenvalues as the function of factor extraction order. The first factor explained 27.74% of the total variance demonstrating the unidimensional structure of the QIDS-SR.

**Table 2:** QIDS-SR ratings at baseline.

	**Mean**	**SD**	**Item–total correlation**	**Alpha, if item deleted**
1. Sleep	2.27	0.81	0.35^**^	0.65
2. Sad mood	0.82	0.87	0.41^**^	0.64
3. Appetite/weight	0.73	0.85	0.40^**^	0.64
4. Concentration/decision making	0.99	0.90	0.60^**^	0.59
5. Self-outlook	0.84	0.88	0.48^**^	0.62
6. Thoughts of death or suicide	0.35	0.65	0.39^**^	0.63
7. Involvement	1.16	0.98	0.62^**^	0.59
8. Energy level	1.17	0.93	0.65^**^	0.57
9. Agitation/retardation	1.34	1.03	0.63^**^	0.59
Total score	9.68	4.06	-	-

*QIDS-SR, quick inventory of depressive symptomatology—self-report.*

** *p < 0.01*.

## Discussion

To the best of our knowledge, this was the first study that examined the reliability and validity of the QIDS-SR in depressed Chinese older adults. The QIDS-SR showed acceptable internal consistency and convergent validity, with unidimensional structure in this population.

Consistent with previous findings in depressed adult patients (27, 33), this study found the internal consistency of the QIDS-SR acceptable. The symptoms of concentration/decision, involvement, energy level, and agitation/retardation performed well in terms of item–total correlations (range of correlation coefficients: 0.60–0.65), which is similar to previous findings in China (33) and also confirms the results of an earlier study that validated the original English version of the QIDS-SR for older adults in the US (range of correlation coefficients: 0.45–0.49) (20). In line with findings of several other studies (19, 21, 33), the sleep item showed the lowest correlation (*r* = 0.35) in this study, while sad mood had moderate item–total correlation (*r* = 0.41), which is lower compared to earlier findings in patients with depression (*r* = 0.60) (27), depressed adolescents (*r* = 0.65) (33), and older adults in the US (*r* = 0.52) (20). This is probably because sad mood may not be the typical presentation in older depressed Asian adults substituted by somatic complaints and symptoms (34–36). Additionally, thoughts of death or suicide also showed relatively weaker correlation in this study (*r* = 0.39), which is not consistent with previous findings; this item had high item–total correlation coefficients (*r* = 0.65) in adolescent with mood disorders (33) and had moderate item–total correlation in depressed adult inpatients (*r* = 0.58 and *r* = 0.52) (27, 37). Suicide is a sensitive topic to discuss in older adults, particularly in traditional Chinese societies (37). Older adults tend to harbor passive suicide ideas or plans, which are difficult to ascertain (8, 38, 39). We hypothesize that participants in this study were reluctant to report thoughts of death in the QIDS-SR assessment or to the interviewer. Although the deaths/suicide item–total correlation coefficient was low, this item still needs to be addressed because of its clinical significance (40).

An acceptable convergent validity for QIDS-SR was found in this study. This is consistent with earlier findings in depressed patients with HBV (22) and depressed schizophrenia patients (21), where the MADRS was used as a comparator rating instrument, suggesting that the QIDS-SR has similar ability to assess the severity of depression compared to the MADRS in the older adults. Exploratory factor analysis revealed the unidimensional structure of the QIDS-SR in older adults, which is also consistent with findings obtained in depression (27) or schizophrenia (21). This supports the notion that all domains of the QIDS-SR reflect the severity of depression (22, 33, 37).

Several limitations of the study should be noted. First, this was a cross-sectional study; hence, the test–retest reliability could not be explored. Second, following the literature (21, 22), only older adults with a MADRs of 7 or higher were included, which may bias the findings to an uncertain extent, because the QIDS-SR is assumed to be valid across all severities of depression. Third, the MADRS was the only reference scale. The MADRS is a generic instrument developed for depressive symptoms in any populations, not specifically for older adults. Thus, the use of MADRS might have reduced the convergent validity of the QIDS-SR in depressed older adults. Fourth, the depression was determined with the MADRS and not by a comprehensive diagnostic exercise. Fifth, participants were recruited from public nursing homes, and the sample size was relatively small, which limits the generalizability of findings to other settings, such as patients living in the community alone or with their families. Finally, due to the lack of healthy controls, the discrimination criterion could not be calculated.

In summary, the QIDS-SR showed an acceptable reliability and validity to assess the severity of depressive symptoms in older adults. The QIDS-SR is brief and unidimensional, which could help clinicians easily estimate the presence and severity of depressive symptoms and monitor their changes over time and the effect of antidepressant treatment in depressed older adults.

## Data Availability Statement

The Institutional Review Board of the University of Macau that approved the study prohibits the authors from making the research dataset of clinical studies publicly available. Readers and all interested researchers may contact the corresponding author who will help apply to the Institutional Review Board of the University of Macau for the release of the data.

## Ethics Statement

The studies involving human participants were reviewed and approved by University of the Macau. The patients/participants provided their written informed consent to participate in this study.

## Author Contributions

Y-TX: study design. FW, SL, QZ, XC, J-XL, and YF: collection, analyses, and interpretation of data. RL, GU, and Y-TX: drafting of the manuscript. KS: critical revision of the manuscript. All authors approval of the final version for publication.

## Funding

This study was supported by the National Natural Science Foundation of China (81901368), the Beijing Municipal Science and Technology Commission (Z181100001518005 and Z181100001718124), and the University of Macau (MYRG2019-00066-FHS).

## Conflict of Interest

The authors declare that the research was conducted in the absence of any commercial or financial relationships that could be construed as a potential conflict of interest.

## Publisher's Note

All claims expressed in this article are solely those of the authors and do not necessarily represent those of their affiliated organizations, or those of the publisher, the editors and the reviewers. Any product that may be evaluated in this article, or claim that may be made by its manufacturer, is not guaranteed or endorsed by the publisher.
